# Development of a New Wearable 3D Sensor Node and Innovative Open Classification System for Dairy Cows’ Behavior

**DOI:** 10.3390/ani12111447

**Published:** 2022-06-03

**Authors:** Daniela Lovarelli, Carlo Brandolese, Lisette Leliveld, Alberto Finzi, Elisabetta Riva, Matteo Grotto, Giorgio Provolo

**Affiliations:** 1 Department of Environmental Science and Policy, University of Milan, Via G. Celoria 2, 20133 Milan, Italy; 2 Department of Electronics, Information and Bioengineering, Politecnico di Milano, Via Ponzio 34, 20133 Milan, Italy; carlo.brandolese@polimi.it; 3 Department of Agricultural and Environmental Sciences, University of Milan, Via G. Celoria 2, 20133 Milan, Italy; lisette.leliveld@unimi.it (L.L.); alberto.finzi@unimi.it (A.F.); elisabetta.riva@unimi.it (E.R.); giorgio.provolo@unimi.it (G.P.); 4 IBT Systems, Via Lomellina 33, 20133 Milan, Italy; matteo.grotto@ibtsystems.it

**Keywords:** cattle behavior, decision tree algorithm, model training, Internet of Things, precision livestock farming

## Abstract

**Simple Summary:**

In order to keep dairy cows under satisfactory health and welfare conditions, it is very important to monitor the animals in their living environment. With the support of technology, and, in particular, with the installation of sensors on neck-collars, cow behavior can be adequately monitored, and different behavioral patterns can be classified. In this study, an open and customizable device has been developed to classify the behaviors of dairy cows. The device communicates with a mobile application via Bluetooth to acquire raw data from behavioral observations and via an ad hoc radio channel to send the data from the device to the gateway. After observing 32 cows on 3 farms for a total of 108 h, several machine learning algorithms were trained to classify their behaviors. The decision tree algorithm was found to be the best compromise between complexity and accuracy to classify standing, lying, eating, and ruminating. The open nature of the system enables the addition of other functions (e.g., localization) and the integration with other information sources, e.g., climatic sensors, to provide a more complete picture of cow health and welfare in the barn.

**Abstract:**

Monitoring dairy cattle behavior can improve the detection of health and welfare issues for early interventions. Often commercial sensors do not provide researchers with sufficient raw and open data; therefore, the aim of this study was to develop an open and customizable system to classify cattle behaviors. A 3D accelerometer device and host-board (i.e., sensor node) were embedded in a case and fixed on a dairy cow collar. It was developed to work in two modes: (1) acquisition mode, where a mobile application supported the raw data collection during observations; and (2) operating mode, where data was processed and sent to a gateway and on the cloud. Accelerations were sampled at 25 Hz and behaviors were classified in 10-min windows. Several algorithms were trained with the 108 h of behavioral data acquired from 32 cows on 3 farms, and after evaluating their computational/memory complexity and accuracy, the Decision Tree algorithm was selected. This model detected standing, lying, eating, and ruminating with an average accuracy of 85.12%. The open nature of this system enables for the addition of other functions (e.g., real-time localization of cows) and the integration with other information sources, e.g., microenvironment and air quality sensors, thereby enhancing data processing potential.

## 1. Introduction

Nowadays, the potentialities of technology and Internet of Things (IoT) are hitting the agricultural and livestock sectors. The support of technology and the concepts of Precision Livestock Farming (PLF) have a huge potential for the progress of livestock farming and they can trigger efficiency improvements, wastes reduction, and environmental sustainability improvements [1]. Similarly, IoT can transfer and store huge amounts of data collected by on-farm sensors [2]. This facilitates data processing and knowledge acquisition, and improves the support for decision-making processes and real-time interventions and is, therefore, very promising. Indeed, one of the primary goals of PLF is to use data to support the decision-making process [3,4] and, thereby, ensure farm animal health and welfare. The behavior of dairy cows provides valuable insights in their health and welfare, and, therefore, its monitoring is a key element in PLF and is well documented in the literature [2,5]. Many studies can be found on the use of sensors to monitor cow behavior [6,7,8], including the time they spend in activities such as lying, standing, feeding, ruminating, and grazing [9,10,11,12], or to detect illnesses [13], estrus events [14,15], or heat stress [16,17]. Different types of wearable accelerometers are available and can be mounted on cows to monitor their behavior; the most common are mounted on legs or on neck-collars [18], while some alternatives are placed on the back [19] or on ears [20]. Whereas many studies have focused on detecting single behaviors with high accuracy [8,21,22,23], comparatively few attempts have been made to classify multiple behaviors [24]. Capturing the main behaviors of dairy cattle provides more insight into their daily activity patterns. The daily time budget is indeed the most informative behavioral measure for evaluating the health and welfare status of dairy cows [25] and the effect of the barn microenvironment on cow behavior [26,27]. Therefore, efforts to estimate the daily time budget through classifying as many behaviors as possible, would result in improved cow welfare monitoring.

For improving the categorization and classification capabilities of sensors, much research has been conducted using the advanced statistics of machine learning and deep learning. Frequently, support vector machine algorithms [28] and decision tree algorithms [19,24] are used, which can achieve high classification accuracies (>80–90% accuracy). Interestingly, Arablouei et al. [21] developed a tri-axial accelerometer that classified the behaviors directly on the device to avoid the post-hoc analysis, thereby reducing computational complexity.

Although many advancements have been made in the field of PLF and IoT, some improvements are still needed for adapting sensors and IoT technology to on-farm monitoring. The collection and storage of big data, data compression and interpretation [21], the simplification of sensor’ structures and of behavioral classification algorithms, and the identification of the most adequate communication channels for rural areas (Bluetooth, LoRa, ZigBee, etc. that are Low Power Wide Area Networks—LPWANs) [29] are among the most important aspects to enhance. In addition, efforts have focused on avoiding false positive and false negative alerts, extending the battery lifespan, and increasing the amount and frequency of data to be stored [8,14,30]. Finally, making systems open can increase the potential of sensors, thereby satisfying a larger group of users. Commercial systems often include black boxes (systems of which the input and output are known, but not their internal functioning) and this complicates the data use and validation [18,23], especially for research purposes. Moreover, commercial devices usually prevent to stream high frequency acceleration data and only provide lumped information on the behavior, typically every few hours. In addition, they are commonly characterized by limited real-time connection possibilities, and only provide data on few behaviors, depending on their installation position [18]. With open systems, the integration of data between different technologies and sensors could increase significantly, allowing to create large databases with enhanced potential for data processing and decision making.

In this context, the goal of this study was to develop an open and customizable system that permits to evaluate, categorize, and classify a wide variety of cattle behaviors. The aim was to use algorithms with low computational and memory complexity in order to have long lasting sensors on farms with satisfactory behavior classification accuracy. Furthermore, efforts were made to have short intervals for behavior recognition, an extended battery lifespan and a direct classification of behaviors on the device to avoid post-processing and reduce the amount of data to store and send to the gateway. These behavior classification sensors are part of a larger project that is focused on the development of an integrated system for the monitoring of the entire barn environment, thus, also including microenvironmental and air quality aspects. The sensors could also be used for other concurrent functions like the real-time location of cows. The cow behavior sensors were, therefore, also developed to fit within the architecture of this larger system. Furthermore, a cloud dashboard was developed to allow front-end users to monitor the collected data remotely and in real-time, enabling them to check on the barn and the animals 24 h/7 d.

This article describes: (i) the sensor developed for classifying the behavior of dairy cows; (ii) the data collection performed on three dairy cattle farms to train and validate the algorithm; and (iii) the model description and validation.

## 2. Materials and Methods

### 2.1. Device Description

A custom device has been designed based on the dual-channel EFR32BG13 Blue Gecko SiP by Silicon Labs [31], featuring two radio channels with integrated power amplifier and balun and a 40 MHz Cortex M4 core with 512 KB of flash memory and 64 KB of RAM. The selected accelerometer is the Bosch BMA400 ultra-low power MEMS [32] with 1 KB of internal hardware FIFO, an accuracy of 1 mG on a range of ±2 G, and a power consumption of 14.5 μA at full speed.

**Figure 1:**
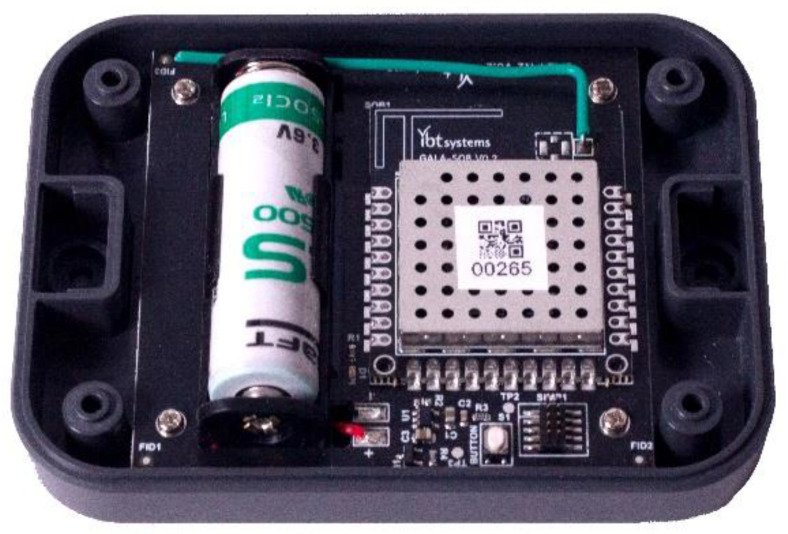
Picture showing the internal section of the sensor node.

The complete device (sensor node) is shown in Figure 1 and is based on a 35 × 45 mm System-on-board (SoB) integrating the components just described and a host-board housing the power supply circuitry and the battery holder. This decoupling choice has been made because this device is just one of a larger family of sensors, which are included in an integrated system, for monitoring a variety of aspects in a dairy cow farm. The complete system (host, SoB, and antennas) is mounted in a 100 × 75 × 22 mm plastic case with IP67 rating.

**Figure 2:**
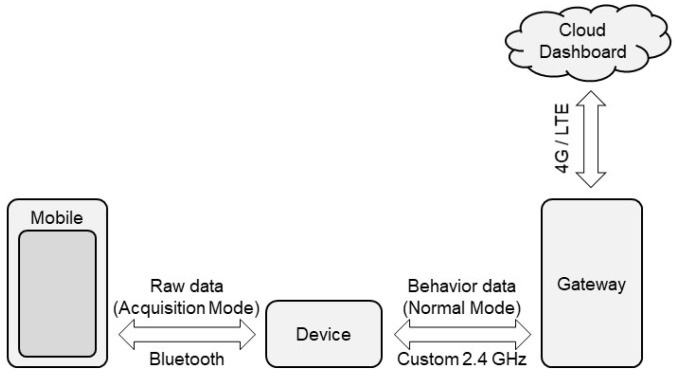
Schematic view of the communication system and the two operating modes (the acquisition mode on the left side and the normal mode on the right side).

This node benefits from a double working mode: it is suited for both raw data acquisition and normal in-field operation. The data acquisition mode is required to collect data on behavioral patterns for training the algorithm and is useful to check the sensor while in operation. Instead, the normal in-field operational mode is aimed at monitoring the behavior of cows continuously and automatically once the algorithm has been developed, implemented in the device firmware, and deployed on the SoB. Figure 2 shows a schematic description of the two operating modes, further detailed in the sections below.

#### 2.1.1. Acquisition Mode

**Figure 3:**
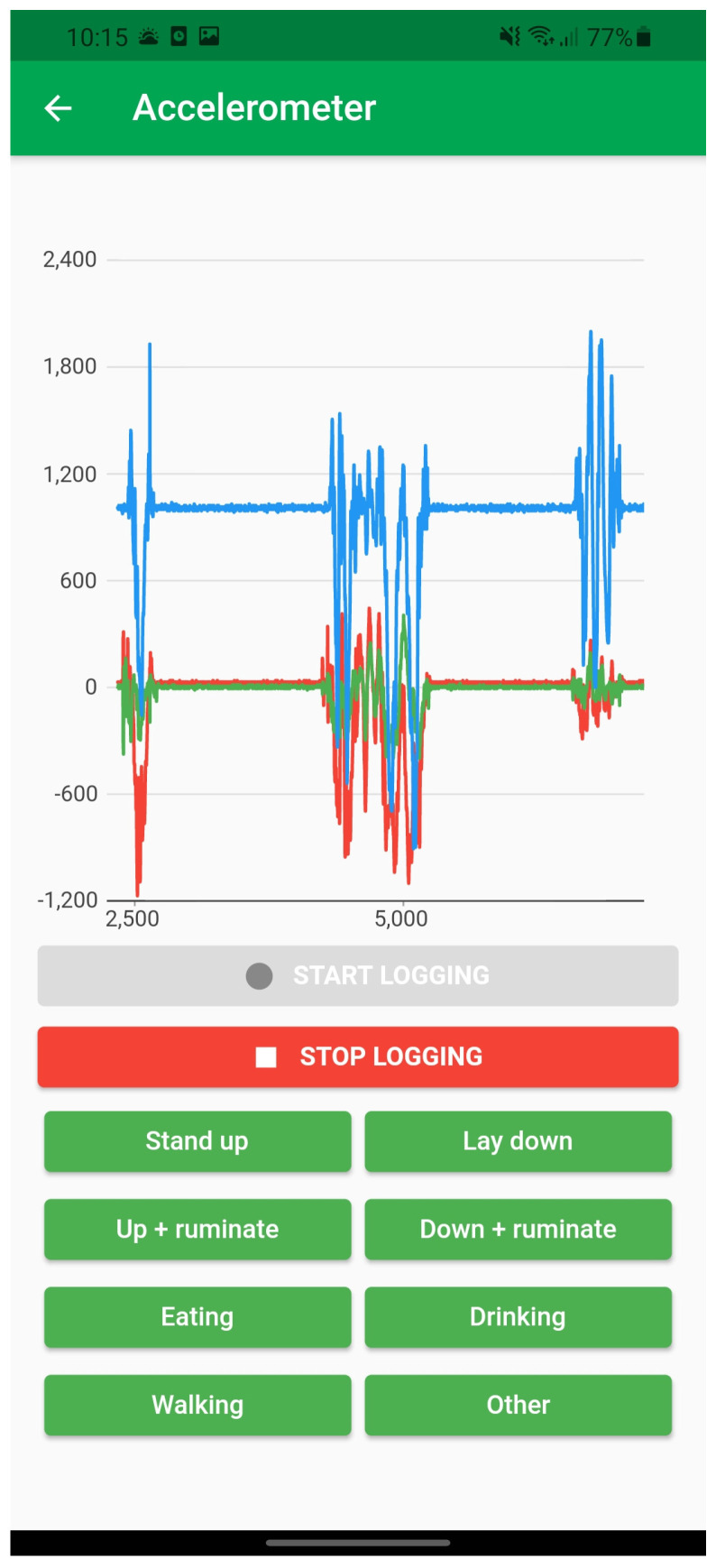
Mobile application for the data acquisition. The upper part shows the real-time 3-axis plot and the bottom part shows the buttons to be pressed by observers. The “start logging” and “stop logging” buttons can be pressed to start and stop the recording of data and the buttons with the behaviors (stand up, up + ruminate, eating, walking, lay down, down + ruminate, drinking, other) can be pressed to enter the behavior classification.

While in the acquisition mode, the sensor node streams data over a Bluetooth channel to a mobile application that has been designed for this purpose (Figure 3). The application plots the data in real time and provides a set of buttons indicating the behaviors to be identified (e.g., standing, lying, ruminating, walking, etc.). By pressing one of the buttons, an identifier indicating the behavior is injected into the real-time stream of acceleration. This results in a “decorated” acceleration time series constituted by tuples of the form $\left(X_{t},Y_{t},Z_{t},\mathrm{and}\;B_{t}\right)$ where $X_{t},\;Y_{t},$ and $Z_{t}$ indicate the accelerations and $B_{t}$ the behavior. This requires the presence of a trained observer that classifies the behavior and enters it in this mobile application.

Due to this operating mode and the interaction with the mobile application, the data collection is simplified, and its reliability improved. It is worth noting, though, that this operating mode requires a significant communication bandwidth (approximately 1.6 kbit s^−1^) which, in turn, implies a continuous Bluetooth connection with the mobile application, leading to a relatively high-power consumption (up to 1 mA, depending on the output transmission power). On a battery-operated device, with a 3.6 V, 2450 mAh lithium-thionyl-chloride primary cell, this power consumption corresponds to less than 3 months of operation, which is suited for data acquisition, but unacceptable for normal operation.

#### 2.1.2. Normal Operating Mode

During the normal operating mode, the device does not use the Bluetooth communication channel but an ad-hoc 2.4 GHz radio channel with a custom lightweight time-slotted protocol for the communication with a gateway, also designed for this purpose. Although it does not transmit, the device advertises on the Bluetooth channel to make it reachable for configuration and for over-the-air firmware updates, again through the mobile application.

The data acquisition, processing, and classification in the normal mode is performed on the device as shown in Figure 2, and the device interacts directly with the gateway through the custom 2.4 GHz channel. The results of the classification are sent to the gateway. Since the processed information has a size of approximately 50 bytes, and the radio channel exhibits a (maximum) bandwidth of 38.4 kbit/s, the entire information can be transmitted in a burst of 10–15 ms, with a sending frequency of 10 min. For the remaining time, the 2.4 GHz transceiver can be switched off. This approach ensures an extremely low power consumption, in the order of 65 μA, corresponding to a maximum lifetime of approximately 3 years.

### 2.2. Data Collection on Farms

#### 2.2.1. Farms Description

The data collection through the acquisition mode of the sensor nodes was conducted on three dairy cattle farms located in Northern Italy, in the province of Cremona (Lombardy region). The area is part of the Po Valley, which is a vast flat area that is highly urbanized and has a high livestock intensity. The monitored barns host Italian Holstein dairy cows in a loose-housing system with free stalls and straw or solid digestate as litter. In the first barn, the monitored building is oriented NE–SW on the long side of the building, with the feeding alley located on the SW side. There are shading curtains to protect the feed and the animals from solar radiation. The barn structure is relatively old and has openings on all sides, a roof with insulating materials and a ridge opening. The barn is equipped with a forced ventilation system above the lying area and with sprinklers above the feeding area (the latter were installed in July 2021). The monitored section of the barn has three lines of cubicles, a total area of 808 m^2^ and hosts about 90 lactating cows. Cows are milked twice a day (at 8 a.m. and 8 p.m.) and the whole milking routine lasts about 2 h. The feed is distributed once a day, around 8.30 a.m.

The second barn is oriented NE–SW on the long side, with the feeding alley SW-oriented. There are moving shading curtains to protect the feed and the animals from solar radiation. The barn was built in 2018. It is fully open on all sides and has a ridge opening and insulation materials on the roof. The barn is equipped with a forced ventilation system above the lying area and sprinklers above the feeding area. The monitored section has two lines of cubicles and a total area of 2121 m^2^. On average, 145 lactating cows are housed in this area. They are milked twice a day (at 4 a.m. and 4 p.m.) with a milking routine that lasts about 1 h per session. The feed is distributed twice a day, at 9 a.m. and 5 p.m.

The third farm has a NW–SE orientation on the long side, with a feeding alley located on the NW side. There are lateral walls on the side of the feed alley and on the two short sides. The barn is equipped with a forced ventilation system above the lying area and with sprinklers above the feeding area. The monitored section of the barn has two lines of cubicles and a total area of 1785 m^2^. About 120 lactating cows are housed in this area. They are milked twice a day (at 3 a.m. and 3 p.m.) with a milking routine that lasts about 1 h per session. The feed is distributed twice a day from May to September (at 7 a.m. and 6 p.m.) and once a day in the colder season (from October to April, 7 a.m.).

#### 2.2.2. Installation of the Sensor Nodes and Behavioral Observations

**Figure 4:**
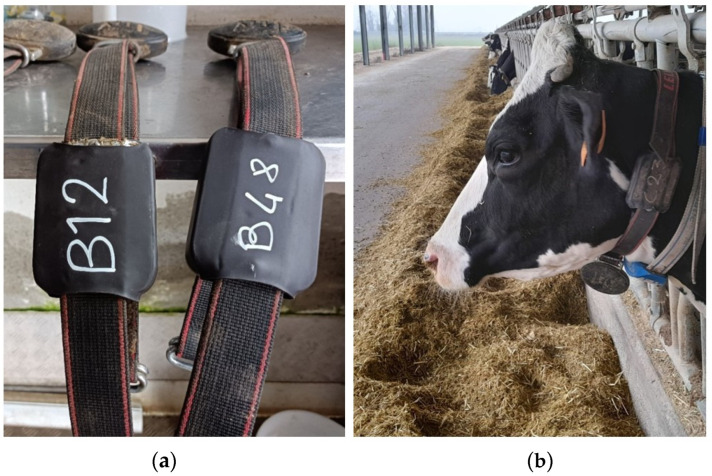
(**a**,**b**). Two sensor nodes mounted on two neck-collars (with the identification code written by pen) (**a**) and the position of the collar on the neck of a cow (**b**).

For the data collection, i.e., behavioral observations, two prototype sensor nodes were built and fixed on two neck collars. These collars had a weight at the bottom to keep the nodes in place. The node was fixed with screws and protecting tape on the upper right part of the collar, at about one third of its length. This position was chosen to allow detecting the movements of the dairy cows also during ingestion-related behaviors (i.e., ruminating, feeding, drinking). These collars were mounted on two cows at a time that were randomly selected from the herd on the day of observation. Figure 4a shows the sensor nodes fixed on the collars and Figure 4b shows the positioning of the collar on the neck of a dairy cow.

For the data collection, continuous behavioral observations were performed by trained observers who scored the behavior of the cows both on a manual protocol and on the developed mobile application. The list of behavior categories is reported in Table 1.

In detail, the manual protocol included the registration of the cow identification number (cow ID), the date and name of the observer, plus the exact time of the day and the indication of the observed behavior (see Table 1). A blank space for notes was also available. At the same time, all behaviors were registered on the mobile application by clicking on the appropriate button for the behavior classification, which allowed to match the behavioral observation with the accelerometer measurements, as described in Section 2.1.1. This was done immediately when the observer noted a change in behavior. Each cow was followed by one observer for 2 to 6 h (until the cow had shown all behaviors of interest). At the end of the day, the collars were removed. The same operation was replicated for 22 days, until data was collected from 32 different cows in total from the three farms (18, 6, and 8 cows per farm, respectively). This process of data collection allowed training the algorithm with a variety of animals and in different farm and management conditions. In total, 108 h of observations of behavior patterns were collected.

### 2.3. Development of the Algorithm

#### 2.3.1. Methodological Approach

**Figure 5:**
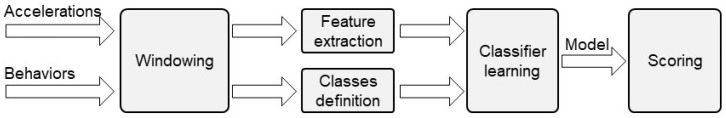
Methodological flow for the development and validation of the model for behavior identification.

The methodology adopted for the behavior identification is structured into several steps as shown in Figure 5 and described in the following paragraphs. After the collection of data (accelerations and behaviors), the steps were: windowing, feature extraction (for accelerations), class definition (for behaviors), classifier learning, and then scoring through the developed and validated model.

The acceleration data from the acquisition mode are sampled at 25 Hz, with a resolution of 12 bit on a range of ±2 G, i.e., with a resolution of 1 mG. Associated to the accelerometric data, the algorithm takes as input the classification of the behaviors observed during the data collection, as described in Section 2.2.

**Figure 6:**
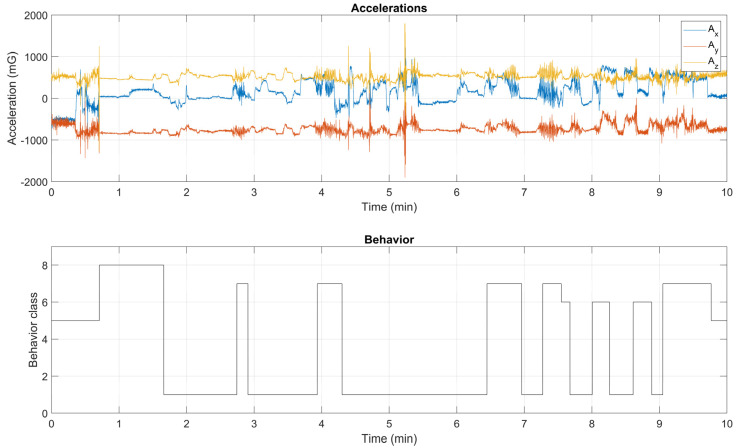
Example of the collected data. Accelerations on the three axes (**top**) and encoded observed behavior (**bottom**).

Therefore, the accelerations and behaviors that have been collected synchronously using the custom sensor node and the mobile application have been combined, as shown in an example in Figure 6. The top plot shows the accelerations in mG on the three axes over time and the bottom plot reports the (encoded) behavior, resulting from the observation. On the *Y*-axis, the behavior classes from 1 to 8 (see Table 1 for the detailed description) are reported.

#### 2.3.2. Behavior Windowing and Class Definition

From here on, the input acceleration data will be indicated as $A_{t}=(X_{t},Y_{t},\;Z_{t})$ and expressed in units of G and the raw behavior data will be indicated as $B_{t}$ and expressed as a discrete variable. The behaviors $\left(B_{1},B_{2},\;\ldots ,\;B_{8}\right)$ that have been considered—and encoded with the integers 1 to 8—are reported in Table 1, i.e.: standing (1), lying (2), standing and ruminating (3), lying and ruminating (4), eating (5), drinking (6), walking (7), and other (8).

Since the behavior of the cows changes much more slowly with respect to the frequency at which the acceleration is sampled, it was decided to split the signals in fixed-length observation windows. However, deciding the duration of the windows is not trivial and can only be conducted experimentally, trading off the granularity of the observations that should be modeled and the accuracy and reliability of the estimation model. Different durations have been considered, namely, 5, 10, 20, 30, and 60 min. Experimental results showed that the best tradeoffs are obtained for 10 and 20 min. In these cases, the distributions of accelerations associated to the different behaviors are well distinguishable and, at the same time, the time resolution of the model is quite detailed. In fact, it is 3 to 12 times finer grained than similar commercial systems. The finer grained model, with a windowing of 10 min, has finally been preferred.

While dynamic and statistical features of the acceleration signals can be easily defined and computed, the association of one single, prevailing behavior seems to be too simplistic. For this reason, a more complex criterion has been adopted. First, for each window, behavior-frequency pairs $\left(b_{i},f_{i}\right)$ are computed and sorted in descending frequency order, i.e.,:

$$\left[\left(b_{1},f_{1}\right),\;\left(b_{2},f_{2}\right),\;\ldots ,\;\left(b_{8},f_{8}\right)\right]$$

where $b_{1}$ is the most frequent behavior and $b_{8}$ is the least frequent. It was decided to characterize a window with two classes, $C_{1}$ and $C_{2}$, associated with the two most relevant behaviors, according to the following rules. The first class is always defined as the most frequent, thus:

$$C_{1}=b_{1}$$

The second class, if appearing with a “significant” frequency (i.e., >40% of the time), is chosen to be $b_{2}$. However, if this class is not frequent enough, it is ignored. The second class is defined as:

$$C_{2}=\left\{\begin{array}{cc} b_{2} & f_{2}\geq f_{min}\\ b_{1} & f_{2}<f_{min} \end{array}\right.$$

where $f_{min}$ defines the minimum frequency of a behavior to be considered significant. This approach leads to 64 classes that can be associated to each window, namely:

$$\left(1,1\right),\;\left(1,2\right),\;\ldots ,\;\left(1,8\right),\;\left(2,1\right),\;\left(2,2\right),\;\ldots ,\;\left(8,8\right)$$

Although much more representative of the actual behavior, a classification into 64 classes is extremely complex and would require a huge number of observations and probably lead to rather complex models. It is important, though, to highlight that the distribution of behaviors is not uniform, as the daily time budget of cows is predominantly occupied by some of the behaviors, in particular lying, eating and ruminating [25].

**Figure 7:**
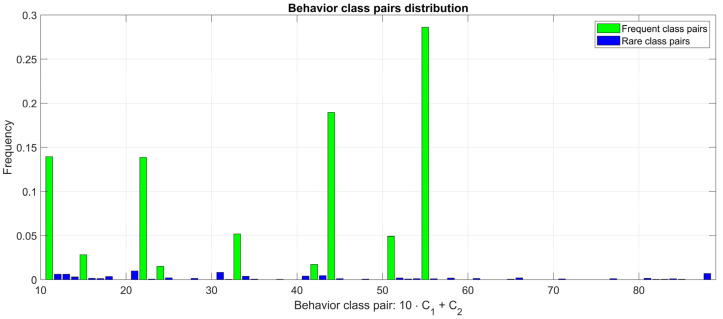
Class pairs frequency.

Secondly, the distribution of the 64 combined classes shows that during a single window, cows tend to show a single behavior with a much higher probability than two different behaviors. Figure 7 shows such a distribution where the frequency $f\left(C_{1},C_{2}\right)$ of the class $\left(C_{1},C_{2}\right)$ is represented at the point of the *x*-axis encoded as $10\cdot C_{1}+C_{2}$.

Since some class pairs are visibly rare (frequency less than 0.01), a “lumped” version of the classes can be defined as:

$$\left({\hat{C}}_{1},{\hat{C}}_{2}\right)=\left\{\begin{array}{cc} \left(8,8\right) & C_{1}\geq 6\\ \left(C_{1},8\right) & f_{\left(C_{1},C_{2}\right)}<f_{low}\\ \left(C_{1},C_{2}\right) & otherwise \end{array}\right.$$

where $f_{low}\;$= 0.01.

This results in the following reduced set of lumped classes:

-
$\left(C_{1},C_{1}\right),\;\left(C_{1},C_{5}\right),\;\left(C_{1},C_{8}\right)$

-
$\left(C_{2},C_{2}\right),\;\left(C_{2},C_{4}\right),\;\left(C_{2},C_{8}\right)$

-
$\left(C_{3},C_{3}\right),\;\left(C_{3},C_{8}\right)$

-
$\left(C_{4},C_{2}\right),\;\left(C_{4},C_{4}\right),\;\left(C_{4},C_{8}\right)$

-
$\left(C_{5},C_{1}\right),\;\left(C_{5},C_{5}\right),\;\left(C_{5},C_{8}\right)$

-
$\left(C_{8},C_{8}\right)$

Following these criteria, the behavior pairs including drinking (6), walking (7), and others (8) cannot be distinguished in the available dataset.

According to the criteria described above and assuming the probability $f_{min}\;$=0.6, a classification $\left(C_{1},C_{1}\right)$ is interpreted as follows:

- $C_{1}$ for at least 60% of the time, certainly
- $C_{1}$ for at least 80% of the time with a probability of 68%
- $C_{1}$ for at least 90% of the time with a probability of 55%
- $C_{1}$ for at least 99% of the time with a probability of 39%
- and a classification $\left(C_{1},C_{2}\right)$ shall be interpreted as follows:
- $C_{2}$ for at least 40% of the time
- $C_{1}$ for a longer time than $C_{2}$

#### 2.3.3. Feature Extraction

##### Acceleration Windowing

As discussed in the previous paragraph, a time window $T_{w}$ of 10 min has been chosen as a good compromise between the granularity of cow behavior over time and the estimation accuracy.

**Figure 8:**
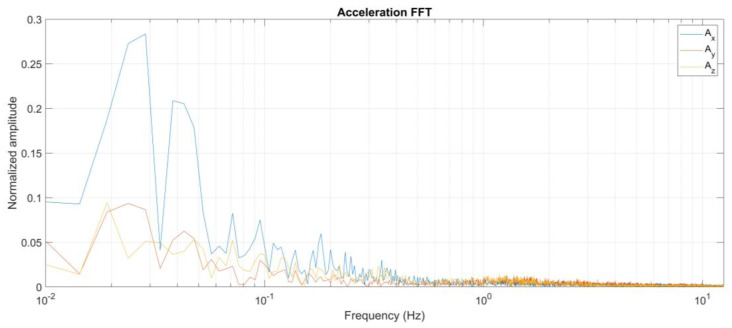
Frequency domain analysis of the acceleration signal on the tree axes (X, Y, Z) calculated by Fast Fourier Transform.

The sampling frequency adopted for the accelerations is $f_{s}=25\;\mathrm{Hz}$ is much higher than the significant dynamics of the animals, because these were found to fall approximately below 5 Hz during some preliminary tests, as is shown in the analysis in the frequency domain reported in Figure 8.

Each of these windows is, therefore, composed of 25⋅60⋅10=15,000 triaxial samples $A_{t}=\left(X_{t},Y_{t},Z_{t}\right)$ with $t=\left[0;14,999\right]$.

Based on previous experience in acceleration signal analysis, a set of 23 features has been defined and computed for each window using Matlab software [33]. However, computing the features on the raw input data will lead to values that are strongly influenced by “noise”, i.e., by accidental accelerations due to the poor fixing of the sensor with respect to the body of the animal and to all unpredictable and unwanted movements due to contact with structural elements of the barn or with other animals. To strongly reduce these effects, accelerations have been processed in “sub-windows” of a duration of $T_{sw}=5$ s according to the following equations. Firstly, average $\left(A_{AVE,k}\right)$, average of the absolute value $\left(A_{ABS,k}\right)$, and standard deviation $\left(A_{STD,k}\right)$ of the acceleration per each axis and per each sub-window have been calculated as:

$$A_{AVE,k}=\frac{1}{f_{s}\cdot T_{sw}}\sum_{t=f_{s}\cdot T_{sw}\cdot k}^{f_{s}\cdot T_{sw}\cdot \left(k+1\right)-1}A_{t}\;A_{ABS,k}=\frac{1}{f_{s}\cdot T_{sw}}\sum_{t=f_{s}\cdot T_{sw}\cdot k}^{f_{s}\cdot T_{sw}\cdot \left(k+1\right)-1}|A_{t}|\;A_{STD,k}={\left[\frac{1}{f_{s}\cdot T_{sw}}\sum_{t=f_{s}\cdot T_{sw}\cdot k}^{f_{s}\cdot T_{sw}\cdot \left(k+1\right)-1}{\left(A_{t}-A_{AVE,k}\right)}^{2}\right]}^{1/2}$$

With $k=\left[0;\frac{T_{w}}{T_{sw}}-1\right]=\left[0;s-1]=[0;119\right]$. This first step leads to three new timeseries per each 10-minute window constituted by $s=T_{w}/T_{sw}=600/5=120$ samples. Then, each windowed measure has been normalized as $\left(A_{N,\;AVE,k}\right):$

$$A_{N,AVE,k}=\frac{A_{AVE,k}}{\underset{k=\left[0;s-1\right]}{\max}|A_{AVE,k}|}$$

And similarly for the other series $A_{ABS,k}$ and $A_{STD,k}$.

The new three three-dimensional series, i.e., these nine series, are the starting point for the statistical features defined for classification. Two statistical features, the generic scalar component of any of the above normalized timeseries (indicated with $V_{N,k}$) and the value of the corresponding, non-normalized time series (indicated with $V_{k}$), are computed as:

$$V_{STD}={\left[\frac{1}{s}\sum_{k=0}^{s-1}V_{k}^{2}\;\right]}^{1/2}\;\;V_{MAX}=\underset{k=\left[0;s-1\right]}{\max}|V_{k}|$$

where $V_{STD}$ stands for the standard deviation of the scalar component V and $V_{MAX}$ is the maximum value of V. This process leads to the definition of a total of 18 features.

In addition to these features, the dynamic features Vector Body Dynamic Acceleration (VeBDA) and Overall Body Dynamic Acceleration (OBDA) have been also computed:

$$VeBDA=\frac{1}{s}\sum_{k=0}^{S-1}{\left|\left|A_{AVE,k}\right|\right|}^{2}\;OBDA=\frac{1}{s}\sum_{k=0}^{S-1}\left|A_{AVE,k}-{\bar{A}}_{AVE}\right|$$

Three other features have finally been considered relevant and potentially representative of periodic behaviors such as walking, ruminating and eating: the correlation coefficients among all pairs of axes (X, Y and Z), namely, $\rho_{XY}=\rho \left(X_{N,AVE,k},Y_{N,AVE,k}\right)$, $\rho_{XZ}=\rho \left(X_{N,AVE,k},Z_{N,AVE,k}\right)$ and $\rho_{YZ}=\rho \left(Y_{N,AVE,k},Z_{N,AVE,k}\right)$, where the correlation coefficients are defined as the diagonal element of the cross-correlation matrix:

$$\rho \left(A,B\right)=\frac{\mathrm{cov}\left(A,B\right)}{\sigma_{A}\sigma_{b}}$$

In conclusion, 23 features have been considered and computed for each time-window.

##### Features Reduction

**Figure 9:**
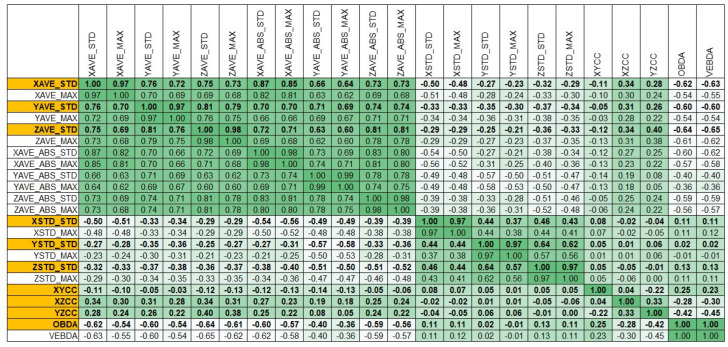
Features cross-correlation matrix.

The features described above are all suggested by the nature of the phenomenon, but their statistical independence needed to be evaluated to reduce the computation complexity of the problem and to avoid overfitting in the model learning phase. Therefore, a cross-correlation matrix, was computed and features with a correlation index above 0.75 were discarded. The cross-correlation matrix is reported in Figure 9. The 10 features indicated in yellow survived the reduction process. When more options were available for choosing sets of features, symmetry, and homogeneity were favored.

It is worth noting that, as an a posteriori confirmation of the correctness of the feature selection approach, the considered models were trained both with the full set and the reduced set, obtaining an average accuracy of 86.4% in the former case and 85.7% in the latter. Since this difference in the resulted accuracy was very small, the reduced set can be used, benefitting simplicity, without losing accuracy.

#### 2.3.4. Classifier Learning

The classification problem of each single window can be expressed as:

$$C=M\left(F\right)=M\left(F_{1},F_{2},\;\ldots ,\;F_{10}\right)$$

where $F=\left[F_{1},F_{2},\;\ldots ,\;F_{10}\right]$ is the vector of the features associated to a certain 10-min window and $C=\left\{1,\;\ldots ,8\right\}$ is the class estimated by the model *M*. It is worth recalling that the adopted original eight behavior classes do not always express disjoint behaviors but are to some extent overlapping, as is the case of behaviors 1 and 3 (Standing, and Standing and ruminating). To account for this circumstance, and, at the same time, to improve the stability of the estimation, four different “class sets” have been defined and used to train four different “basic classifiers”, namely:

- Classifier 1:$\;S_{1}=\left\{\;1,\;2,\;3,\;4,\;5,\;6,\;7,\;8\;\right\}$
- Classifier 2: $S_{2}=\left\{\;\left(1,\;2\right),\left(3,\;4\right),\;5,\;\left(6,\;7,\;8\right)\;\right\}$
- Classifier 3: $S_{3}=\left\{\;\left(1,\;3\right),\;\left(2,\;4\right),\;5,\;\left(6,\;7,\;8\right)\;\right\}$
- Classifier 4: $S_{4}=\left\{\;\left(1,\;2,\;3,\;4\right),\;5,\;\left(6,\;7,\;8\right)\;\right\}$

Classifier 1 is the finer-grained classifier and potentially distinguishes all behavior classes (1 to 8); classifier 2 distinguishes between non-ruminating (1, 2), ruminating (3, 4), eating (5), and other behavior (6, 7, 8); classifier 3 distinguishes between standing (1, 3), lying (2, 4), eating (5), and other behavior (6, 7, 8); and, finally, classifier 4 distinguishes between eating (5), non-eating (1, 2, 3, 4), and other behaviors (6, 7, 8).

In addition to these basic classifiers, a class pair classifier model has also been trained. This classifier named S_5 distinguishes the class pairs defined in Section 2.3.1, that is:

- Classifier 5: $S_{5}=\left\{11,\;15,\;18,\;22,\;24,\;28,\;33,\;38,\;42,\;44,\;48,\;51,\;55,\;58,\;88\right\}$

where each element indicates the two most frequent behaviors (indicated by coupling behaviors 1–8) in a time window, in decreasing order.

**Figure 10:**
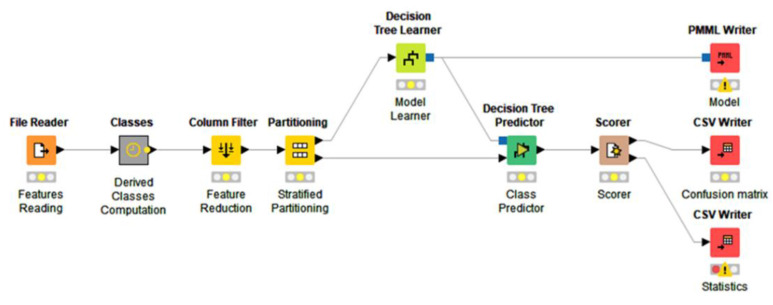
Knime workflow.

The adopted learning procedure is based on Knime software [34] and is structured as shown in the workflow reported in Figure 10. Firstly, all the features computed with Matlab are loaded in the workflow, along with the actual behavior classifications $S_{1}$ and $S_{5}$; then each vector of the dataset is enriched with the derived classes $S_{2},\ldots ,\;S_{4}$ computed starting from the classes in $S_{1}$. A column filter is then applied to the input vectors to remove unnecessary features according to the analysis described above.

The dataset of all the reduced vectors is then fed to a partitioning node which divides it into a learning set (upper path in Figure 10) and a test set (lower path in Figure 10) according to a learning/test ratio of 75%/25% with stratified sampling with respect to the actual behavior class. The learning set is then passed to the specific learner node (a decision tree in the example of Figure 10) while the test set is passed to the predictor node, which performs classification according to the model generated by the learner. Finally, the predictions are evaluated by a scorer node and the results of the workflow, i.e., the model in PMML format, the confusion matrix and the accuracy statistics, are saved to files for further analyses.

#### 2.3.5. Combined Classifier

Decision tree models were trained for each of the five classifiers $S_{i}$ leading to five different estimates $s_{1}\in S_{1}$, $s_{2}\in S_{2}$, $\ldots ,\;s_{5}\in S_{5}$ of the actual behavior c∈C for each 10-min time window. To combine accuracy and specificity, a new estimator $S_{6}$ has been defined a-posteriori as the weighted combination of the five basic estimators according to the following procedure. Let $w_{i}$ be the weight associated to the classifier $S_{i}$. For each class $k\in \left\{1,\;\ldots ,\;8\right\}$ the overall class weight $W\left(k\right)$ is defined as the sum of the weight of each classifier whose result contains the class k, that is:

$$W\left(k\right)=\sum_{i=1}^{5}\omega_{i,k}$$

where:

$$\omega_{i,k}=\{\begin{array}{cc} w_{i} & if\;\;k\in s_{i}\\ 0 & otherwise \end{array}$$

The result of the new combined classifier $S_{6}$ is then defined as the class k whose overall weight $W\left(k\right)$ is maximum, that is:

$$S_{6}=k\;|\;W\left(k\right)=\underset{k}{\max}W\left(k\right)$$

#### 2.3.6. Model Validation

For the learning phase, a subset of all feature vectors was used, while the remaining vectors were used for the testing. The overall number of feature vectors is 19,524, of which 75% were used for training and the remaining 25% for validation.

To select the models to be adopted, two crucial aspects were considered: the complexity of application in terms of data memory and code memory requirements and the model accuracy. The trained model, in fact, needs to be implemented on the sensor node, which is a tiny microcontroller with less than 64 KB of flash memory and approximately 8 KB of RAM memory available and delivering less than 100 MIPS.

Finally, a sensitivity analysis was carried out to confirm the adequateness of the partitioning of the dataset for the model training and validation.

## 3. Results

### 3.1. Observed Behaviors and Evaluations about the Feature Selection

**Figure 11:**
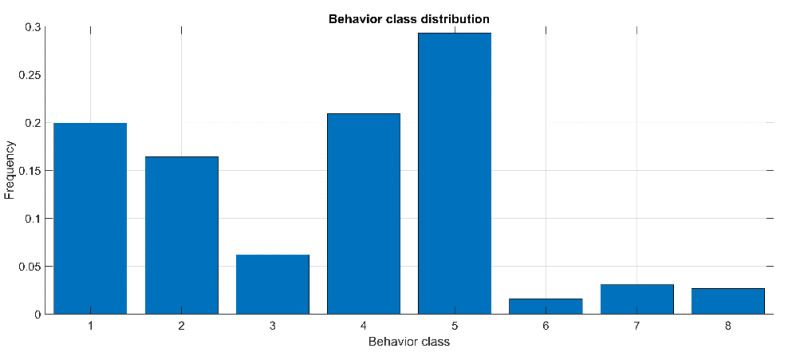
Results of the class frequency distribution from the observed dairy cows. Numbers on the *x*-axis refer to the behavior classes: 1 = standing; 2 = lying; 3 = standing and ruminating; 4 = lying and ruminating; 5 = eating; 6 = drinking; 7 = walking; 8 = other.

Figure 11 shows the distribution of observed behaviors from the 32 monitored cows, obtained by analyzing more than 100 h of observations. It can be noticed that the behavior classes are far from being uniform, with classes 6 (drinking), 7 (walking), and 8 (other) having very low frequencies (<5%).

This result can be in part attributed to the part of the day in which the observations took place; however, it is consistent with the average daily time budget of cows from the literature [25] and with the fact that transitional behaviors such as walking, standing up, or lying down, as well as other social or maintenance behaviors like drinking, are less frequent and of short duration [18], making their identification through sensors and algorithms quite complex. In this study, behavior observations were collected from 32 cows in 3 farms; therefore, there was the influence of different farm management organization. This variability, which is larger than in the majority of studies present in literature (i.e., less than 10% of studies used more than 30 cows, according to the findings by Riaboff et al. [18]), can have negatively influenced the accuracy of the algorithm while increasing its robustness. Moreover, more than 40 h of behavioral observations are highly recommended in view of robust predictions [35].

In this study, 108 h of behavioral observations from 32 cows were used, therefore, the reported findings can be considered sufficiently robust. Moreover, the variability (three farms) and size of the behavioral data ensures that the developed sensors can be widely applicable. This is well above the minimum recommendation for optimal framework for achieving good prediction performances using accelerometers (2 farms, 25 animals, 40 h) as reported by Riaboff et al. [18].

### 3.2. Training and Validation of the Model

Regarding the results of the model selection, several algorithms were trained and evaluated with respect to accuracy and complexity. These assessment results are summarized in Table 2.

Among the evaluated algorithms, 3 had a low complexity in terms of computational and memory requirements: Decision Tree, Multi-Layer Perceptron, and Probabilistic Neural Networks. Among these, the Decision Tree (77−87%) outperformed the accuracy of Multi-Layer Perceptron (65−79%) and Probabilistic Neural Networks (70−74%) in all four basic classifiers. It presented low complexity and high accuracy; therefore, it was selected. In the Decision Tree algorithm, the overall memory footprint of the code implementing the four classifiers is 46 KB, with fewer than 400 bytes of RAM required. Furthermore, the execution time needed for feature extraction and estimation is less than 500 ms, which is more than acceptable in terms of energy consumption.

Having selected the Decision Tree algorithm, the accuracy of the decision trees trained for the five classifiers (see Section 2.3.4), $S_{1},\ldots ,\;S_{5},$ is calculated and summarized in Table 3. As expected, the accuracies of these estimates are the lowest for classifiers $S_{1}$ (79.9%) and $S_{5}$ (69.3%), which were more specific (Classifier 1 distinguishes all behavior classes and Classifier 5 considers the class pairs); instead, classifier $S_{4}$, being less specific in the classification of the behaviors (i.e., it distinguishes eating, non-eating and other), shows the highest accuracy (91.0%).

Regarding the accuracy of the combined classifier $S_{6}$, two different sets of weights have been used: the first choice assumes all the weights to be equal to 1, while the second uses the accuracies of the basic estimators (reported in Table 3) as weights. For this combined classifier, the results achieved with the decision tree algorithm show an accuracy of 81.00% and 81.05%, respectively, for the two sets of weights. This difference is very small and, therefore, weights equal to 1 have been considered to obtain the classifier $S_{6}$.

### 3.3. Sensitivity Analysis

Although the available dataset is large enough for the selected classifier model, a sensitivity analysis has been performed to verify that a 75%/25% partitioning of the datasets leads to stable models. To this purpose, different sizes of learning and test sets have been used, leading to the results reported in Table 4.

From the table, it can be derived that increasing the size of the learning set above 75% does not improve the accuracy in a relevant way. Therefore, the 75%/25% partitioning was considered as the most adequate.

Though the accuracy of the models is rather good (85.12%), it must be noted that the choice of subdividing time into 10-min windows introduces an effect of quantization with respect to the actual time spent by the cows in each of the considered behaviors.

To evaluate the accuracy over time of the combined classifier $S_{6}$, 1950 time windows from the test set have been considered. For each window, W, the actual time $T\left(W,b_{i}\right)$ spent in a specific behavior, $b_{i},$ is computed as the total number of samples associated with that behavior, multiplied by the sampling period, that is:

$$T\left(W,b_{i}\right)=\Delta t\cdot \sum_{B_{t}\in W}^{}\delta_{B_{t},\;b_{i}}$$

where δ is the Kronecker symbol and $\Delta t=1/f_{s}=40\;\mathrm{ms}$ is the sampling period. On the other hand, the estimated time is simply 10 min for the estimated behavior of the considered window and zero for all other behaviors.

**Figure 12:**
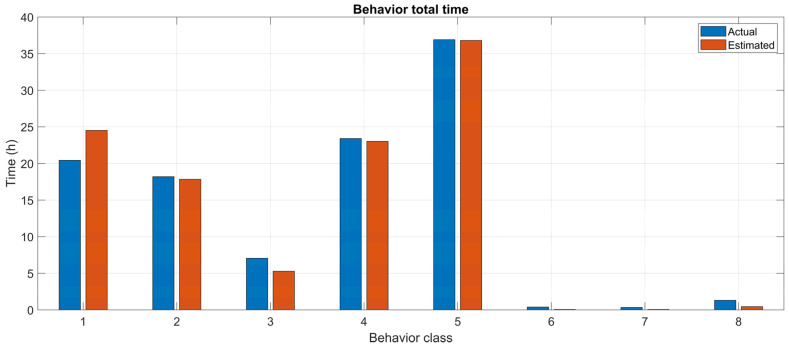
Time accuracy of the combined classifier of actual and estimated behavioral classes. Numbers on the *x*-axis refer to the behavior classes: 1 = standing; 2 = lying; 3 = standing and ruminating; 4 = lying and ruminating; 5 = eating; 6 = drinking; 7 = walking; 8 = other.

The comparisons of the actual and the model-estimated behavioral patterns over time are shown in Figure 12. The relative errors for the significant classes 1, …, 5 (classes 6, 7, and 8 are very rare and not relevant for statistical analysis) are reported in Table 5. Overall, the accuracy over time is 92.45%.

## 4. Discussion

In this study, an open sensor node was developed to classify multiple behavioral patterns, i.e., standing, lying, standing and ruminating, lying and ruminating, eating, and other (including walking and drinking), using one single sensor positioned on a neck collar. This sensor could be used for acquiring data from behavioral observations by using a mobile application working via Bluetooth, as well as for sending processed data to the gateway via a dedicated radio channel. After the acquisition of 108 h of behavioral data from 32 cows in 3 farms, several machine learning algorithms were trained. The Decision Tree algorithm was selected due to its low computational and memory complexity, and due to its satisfactory results in behavior classification, with an accuracy of 85.12%. Although walking and drinking could be encoded, their frequency was too small to be identified by the algorithm. The same issue was raised by Vázquez Diosdado et al. [24] who omitted walking, drinking, and brushing for a similar lack of data. The other classified behaviors, instead, are those to which usually the highest interest is paid. For instance, Riaboff et al. [18] reviewed studies on ruminants’ behaviors classification (including cows, sheep and goats), and found that the most frequently predicted behaviors are eating and grazing (if on pasture) (21.3% of studies), moving (walking, running, and searching, 18.8%), standing (15.9%), and lying (13.0%). In general, most of the studies developed systems that rely on single sensors to classify only a few behaviors with high accuracy (>80–90%), comparable to what commercial sensors are offering. Vázquez Diosdado et al. [24] suggest that when detecting more than a few (e.g., 2–3) behaviors with a single sensor, the prediction accuracy of the models drops substantially. Considering that this system classified several behaviors with a single sensor, the average accuracy of the classification model that has been achieved in this study (85.12%) can be considered relatively high. As mentioned in the introduction, classifying several main behaviors allows a better estimation of the time budget of dairy cows and therefore results in more complete cow welfare monitoring.

The aim was to use the sensor node on the farm for a long period; therefore, the methodological choices were aimed at increasing battery life by reducing the computational and memory complexity (25-Hz sampling frequency, 10-min time windows and a 10-min frequency for sending data from the sensor to the gateway with the ad-hoc 2.4 GHz radio channel). These choices differ from the approaches of other studies, such as of [19,30,45,46] who adopted much shorter time windows and achieved accuracies >90–95%. Robert et al. [30] stated that 5-s time windows provide the best compromise between accuracy and memory constraints. However, due to the need of applying robust sensors that are able to function for a long time on the farm, the 10-min window was preferred here as a better trade-off between model complexity and classification accuracy. A similar approach to this study was adopted by Vázquez Diosdado et al. [24], who classified behavior of 6 cows with a 10-min window and a decision tree approach. Precision and sensitivity were reported, rather than accuracy; therefore, direct comparisons cannot be made. However, they obtained an overall model sensitivity and precision equal to 88% and 82%, respectively (for lying, 77% and 99%; standing, 88% and 55%; and feeding, 99% and 93%). They also highlighted the importance of developing simple behavioral classification algorithms for Precision Livestock Farming (PLF) purposes. This facilitates the computation, model training and validation process, as well as its use. Another similar approach to this study was the one by Martiskainen et al. [28] who adopted support vector machine (SVM) algorithms to identify a series of behaviors. They found an overall accuracy >80% for the classes of standing, lying, ruminating, feeding, walking normally, and lame walking, while a lower accuracy was found for the transitional behaviors of lying down and standing up. However, SVM are much more complex in computation and were therefore disregarded in this study.

Another aspect of this study that needs to be highlighted is that observing behaviors of a large sample of animals (32 cows from 3 farms) and for a long time (108 h) allowed to train a robust model. This sampling dimension is larger than in the major part of studies [18], but it is very important. The accuracy of this decision tree model is, indeed, negatively influenced by the large size and variability of the sample, but, on the other hand, this size and variability also enhances its applicability in different livestock contexts.

The sensor node presented in this study and its conceptual data integration can be interesting for: (1) the PLF purposes of continuous monitoring of single animals or aggregated groups of animals to support the management and decision process of the farmer; (2) remote data processing for long periods, which can allow assessments on animals performances; and (3) finally, since this system is open and is characterized by a low computational and memory complexity, it can become part of a larger integrated monitoring system that can allow building large databases for improved data processing and support to decision making. Such a system could also automatically monitor and regulate systems such as forced ventilation and scrapers in order to prevent undesired conditions. This is a key point raised also by Fournel et al. [47].

In the future, this sensor node can be further improved by enhancing the classification capabilities of behavior classes, such as drinking, estrus, respiration rate, and lame walking, in order to integrate more information in a PLF environment. The sensor node can be used also to identify the cows during milking in order to collect data on milk yield and other parameters, such as milk characteristics and milking time. Linking the node to milk production can give additional information on the relationship between productivity, behavior, and environment. Moreover, due to the open nature of the system and its multiple transmission protocol, a future enhancement of this system could be to integrate the position of cows with indoor real time location systems, giving additional insights on cow welfare. It could, for example, evaluate the preferential use of the different areas of the barn and the aggregation of both milk and beef cattle.

## 5. Conclusions

This study deals with the development of a wearable 3D sensor node and an algorithm based on a Decision Tree to identify a wide series of behaviors of dairy cows, i.e., standing, lying, standing and ruminating, lying and ruminating, eating, and other. This algorithm was trained and validated through different steps of machine learning techniques. It was stored on a node sensor, embedded in a plastic case and then fixed on a dairy cow neck collar. Even though the classification of many behaviors, the large sample size (32 dairy cows in 3 livestock farms), low complexity, and 10-min time window may have lowered the overall accuracy, they all improve the robustness and relevance of the system for on-farm welfare monitoring. The model accuracy for the specific classes is 85.12%, which is comparable with similar studies. The architecture of the sensor nodes and classification system are open and customizable and aimed at a low application complexity and long operation duration on the farm. Although the device has demonstrated to be effective in identifying the main cow behaviors, there is still room for improvement by including other behaviors, such as walking, estrus identification, lame walking, and drinking. The sensor can also be an active part of a real-time geo-location system to determine how dairy or beef cattle use the different areas of the barn. Finally, since this system is designed to be part of a larger system that integrates both behavioral and microenvironmental data, it will enable the automatic regulation of microenvironmental systems to avoid situations of potential health and welfare risks in real time.

## Figures and Tables

**Table 1:** Ethogram of the studied cow behaviors. Class refers to the encoded button that was pushed during observations and to the identification of the behavior during the algorithm development.

Class	Behavior	Description
1	Standing	The cow has at least 3 legs resting without moving the body. It includes head movements and interactions with other animals. There may be small movements that do not significantly change the position, covering less space than the animal’s body length. The cow does not ruminate.
2	Lying	The body is in contact with the bottom of the cubicle. The cow can move its head and interact with other animals. The cow does not ruminate.
3	Standing and ruminating	Like standing, but in addition the cow ruminates. Ruminating: sequence consisting of regurgitating a bolus, followed by chewing the cud and then swallowing the masticated cud.
4	Lying and ruminating	Like lying, but in addition the cow ruminates.
5	Eating	Sequence consisting of lowering the head to the feed, taking a bite, chewing and swallowing. Short interruptions and interactions with other cows may occur.
6	Drinking	The cow has its head in the drinking trough and drinks water.
7	Walking	The cow changes position with a movement in a defined direction, covering at least a space equal to the animal’s body length.
8	Other	Other behaviors that do not fit in any of the previous categories (specification of the behavior was noted down manually).

**Table 2:** Model evaluation algorithms with the related accuracy (percentage of correctly classified 10-min time windows) and complexity of application (computational and memory requirements).

Model	Reference	Accuracy	Complexity
Fuzzy Rules	[36]	87–92%	Unfeasible
Support Vector Machines	[37]		Unfeasible
K-Nearest Neighbors	[38]		Unfeasible
Random Forest (large)	[39]		Critical
Ensemble Decision Tree (large)	[40]		Critical
Random Forest (small)	[39]	75–90%	Medium
Decision Tree	[41]		Low
Multi-Layer Perceptron	[42]	55–75%	Low
Probabilistic Neural Networks	[43]		Low
Naïve Bayes	[44]		Medium

**Table 3:** Model accuracy (percentage of correctly classified 10-min time windows) for the six classifiers.

Classifier	$S_{1}$	$S_{2}$	$S_{3}$	$S_{4}$	$S_{5}$	$S_{6}$
Accuracy	79.9%	87.6%	82.0%	91.0%	69.3%	81.0%

**Table 4:** Model accuracy (percentage of correctly classified 10-min time windows) versus partitioning.

Classifiers	Partitioning			
	25%/75%	50%/50%	75%/25%	90%/10%
Classifier 1	74.6%	76.5%	79.9%	80.0%
Classifier 2	84.4%	85.6%	87.6%	88.0%
Classifier 3	77.3%	80.3%	82.0%	81.9%
Classifier 4	89.3%	89.7%	91.0%	90.2%
Classifier 5	63.2%	66.4%	69.3%	71.0%

**Table 5:** Relative error of the combined classifier for the relevant classes of actual and estimated behavior.

Class Identifier	1	2	3	4	5
Class	Standing	Lying	Standing and ruminating	Lying and ruminating	Eating
Error	−19.94%	1.81%	25.00%	1.65%	0.29%

## Data Availability

The data presented in this study are available on request from the corresponding author.

## References

[1] Lovarelli D., Bacenetti J., Guarino M. (2020). A review on dairy cattle farming: Is precision livestock farming the compromise for an environmental, economic and social sustainable production?. J. Clean. Prod..

[2] Dominiak K.N., Kristensen A.R. (2017). Prioritizing alarms from sensor-based detection models in livestock production—A review on model performance and alarm reducing methods. Comput. Electron. Agric..

[3] Berckmans D. (2017). General introduction to precision livestock farming. Anim. Front..

[4] Halachmi I., Guarino M., Bewley J., Pastell M. (2019). Smart Animal Agriculture: Application of Real-Time Sensors to Improve Animal Well-Being and Production. Annu. Rev. Anim. Biosci..

[5] Leliveld L.M.C., Provolo G. (2020). A review of welfare indicators of indoor-housed dairy cow as a basis for integrated automatic welfare assessment systems. Animals.

[6] Benaissa S., Tuyttens F.A.M., Plets D., de Pessemier T., Trogh J., Tanghe E., Martens L., Vandaele L., Van Nuffel A., Joseph W. (2019). On the use of on-cow accelerometers for the classification of behaviours in dairy barns. Res. Vet. Sci..

[7] Hendriks S.J., Phyn C.V.C., Huzzey J.M., Mueller K.R., Turner S.-A., Donaghy D.J., Roche J.R. (2020). Graduate Student Literature Review: Evaluating the appropriate use of wearable accelerometers in research to monitor lying behaviors of dairy cows. J. Dairy Sci..

[8] Pavlovic D., Davison C., Hamilton A., Marko O., Atkinson R., Michie C., Crnojevic V., Andonovic I., Bellekens X., Tachtatzis C. (2021). Classification of cattle behaviours using neck-mounted accelerometer-equipped collars and convolutional neural networks. Sensors.

[9] Arcidiacono C., Porto S.M.C., Mancino M., Cascone G. (2017). Development of a threshold-based classifier for real-time recognition of cow feeding and standing behavioural activities from accelerometer data. Comput. Electron. Agric..

[10] Carslake C., Vázquez-Diosdado J.A., Kaler J. (2021). Machine learning algorithms to classify and quantify multiple behaviours in dairy calves using a sensor–moving beyond classification in precision livestock. Sensors.

[11] Mottram T. (2016). Animal board invited review: Precision livestock farming for dairy cows with a focus on oestrus detection. Animal.

[12] Riaboff L., Relun A., Petiot C.-E., Feuilloy S., Couvreur S., Madouasse A. (2021). Identification of discriminating behavioural and movement variables in lameness scores of dairy cows at pasture from accelerometer and GPS sensors using a Partial Least Squares Discriminant Analysis. Prev. Vet. Med..

[13] Pastell M., Tiusanen J., Hakojärvi M., Hänninen L. (2009). A wireless accelerometer system with wavelet analysis for assessing lameness in cattle. Biosyst. Eng..

[14] Wang J., Zhang Y., Wang J., Zhao K., Li X., Liu B. (2022). Using machine-learning technique for estrus onset detection in dairy cows from acceleration and location data acquired by a neck-tag. Biosyst. Eng..

[15] Arcidiacono C., Porto S.M.C., Mancino M., Cascone G. (2018). A software tool for the automatic and real-time analysis of cow velocity data in free-stall barns: The case study of oestrus detection from Ultra-Wide-Band data. Biosyst. Eng..

[16] Davison C., Michie C., Hamilton A., Tachtatzis C., Andonovic I., Gilroy M. (2020). Detecting heat stress in dairy cattle using neck-mounted activity collars. Agriculture.

[17] Lovarelli D., Finzi A., Mattachini G., Riva E. (2020). A Survey of Dairy Cattle Behavior in Different Barns in Northern Italy. Animals.

[18] Riaboff L., Shalloo L., Smeaton A.F., Couvreur S., Madouasse A., Keane M.T. (2022). Predicting livestock behaviour using accelerometers: A systematic review of processing techniques for ruminant behaviour prediction from raw accelerometer data. Comput. Electron. Agric..

[19] Achour B., Belkadi M., Aoudjit R., Laghrouche M. (2019). Unsupervised automated monitoring of dairy cows’ behavior based on Inertial Measurement Unit attached to their back. Comput. Electron. Agric..

[20] Krieger S., Oczak M., Lidauer L., Berger A., Kickinger F., Öhlschuster M., Auer W., Drillich M., Iwersen M. (2019). An ear-attached accelerometer as an on-farm device to predict the onset of calving in dairy cows. Biosyst. Eng..

[21] Arablouei R., Currie L., Kusy B., Ingham A., Greenwood P.L., Bishop-Hurley G. (2021). In-situ classification of cattle behavior using accelerometry data. Comput. Electron. Agric..

[22] Barker Z.E., Vázquez Diosdado J.A., Codling E.A., Bell N.J., Hodges H.R., Croft D.P., Amory J.R. (2018). Use of novel sensors combining local positioning and acceleration to measure feeding behavior differences associated with lameness in dairy cattle. J. Dairy Sci..

[23] Bidder O.R., Campbell H.A., Gómez-Laich A., Urgé P., Walker J., Cai Y., Gao L., Quintana F., Wilson R.P. (2014). Love thy neighbour: Automatic animal behavioural classification of acceleration data using the k-nearest neighbour algorithm. PLoS ONE.

[24] Vázquez Diosdado J.A., Barker Z.E., Hodges H.R., Amory J.R., Croft D.P., Bell N.J., Codling E.A. (2015). Classification of behaviour in housed dairy cows using an accelerometer-based activity monitoring system. Anim. Biotelemetry.

[25] Gomez A., Cook N.B. (2010). Time budgets of lactating dairy cattle in commercial freestall herds. J. Dairy Sci..

[26] Collier R.J., Dahl G.E., Vanbaale M.J. (2006). Major advances associated with environmental effects on dairy cattle. J. Dairy Sci..

[27] Das S.K., Karunakaran M., Barbuddhe S.B., Singh N.P. (2015). Effect of Orientation, Ventilation, Floor Space Allowance and Cooling Arrangement on Milk Yield and Microclimate of Dairy Shed in Goa. J. Anim. Res..

[28] Martiskainen P., Järvinen M., Skön J.P., Tiirikainen J., Kolehmainen M., Mononen J. (2009). Cow behaviour pattern recognition using a three-dimensional accelerometer and support vector machines. Appl. Anim. Behav. Sci..

[29] Germani L., Mecarelli V., Baruffa G., Rugini L., Frescura F. (2019). An IoT architecture for continuous livestock monitoring using lora LPWAN. Electronics.

[30] Robert B., White B.J., Renter D.G., Larson R.L. (2009). Evaluation of three-dimensional accelerometers to monitor and classify behavior patterns in cattle. Comput. Electron. Agric..

[31] Silicon Labs. https://www.silabs.com/wireless/bluetooth/efr32bg13-series-1-modules.

[32] Bosch. https://www.bosch-sensortec.com/products/motion-sensors/accelerometers/bma400/.

[33] Matlab, Mathworks. https://www.mathworks.com/products/matlab.html.

[34] Knime. https://www.knime.com.

[35] González L.A., Bishop-Hurley G.J., Handcock R.N., Crossman C. (2015). Behavioral classification of data from collars containing motion sensors in grazing cattle. Comput. Electron. Agric..

[36] Wang L., Mendel J.M. (1992). Generating fuzzy rules by learning from examples. IEEE Trans. Syst. Man Cybern..

[37] Hearst M.A., Dumais S.T., Osuna E., Platt J., Scholkopf B. (1998). Support vector machines. IEEE Intell. Syst. Appl..

[38] Fix E., Hodges J.L. (1951). Discriminatory Analysis. Nonparametric Discrimination: Consistency Properties (Report).

[39] Ho T.K. Random Decision Forests. Proceedings of the 3rd International Conference on Document Analysis and Recognition.

[40] Rokach L. (2009). Ensemble-based classifiers. Artif. Intell. Rev..

[41] Quinlan J.R. (1986). Induction of decision trees. Mach. Learn..

[42] Rumelhart D.E., Hinton G.E., Ronald J.W. (1985). Learning Internal Representations by Error Propagation.

[43] Specht D.F. (1990). Probabilistic neural networks. Neural Netw..

[44] Nir F., Geiger D., Goldszmidt M. (1997). Bayesian network classifiers. Mach. Learn..

[45] Andriamandroso A.L.H., Lebeau F., Beckers Y., Froidmont E., Dufrasne I., Heinesch B., Dumortier P., Blanchy G., Blaise Y., Bindelle J. (2017). Development of an open-source algorithm based on inertial measurement units (IMU) of a smartphone to detect cattle grass intake and ruminating behaviors. Comput. Electron. Agric..

[46] Dutta R., Smith D., Rawnsley R., Bishop-Hurley G., Hills J., Timms G., Henry D. (2015). Dynamic cattle behavioural classification using supervised ensemble classifiers. Comput. Electron. Agric..

[47] Fournel S., Rousseau A.N., Laberge B. (2017). Rethinking environment control strategy of confined animal housing systems through precision livestock farming. Biosyst. Eng..

